# Relational subgraphs fused with complete subgraphs based on the knowledge graph for mining protein complexes

**DOI:** 10.1038/s41598-025-18281-7

**Published:** 2025-10-29

**Authors:** Ruixue Zhao, Dandan Zhang, Yuantao Kou, Guojian Xian, Xiao Yang

**Affiliations:** 1 Agricultural Information Institute of Chinese Academy of Agricultural Sciences, Beijing, 100081 China; 2 Key Laboratory of Agricultural Integration Publishing Knowledge Mining and Knowledge Service, National Press and Publication Administration, Beijing, 100081 China; 3 Key Laboratory of Agricultural Big Data, Ministry of Agriculture and Rural Affairs, Beijing, 100081 China

**Keywords:** Protein complex, Knowledge graph, Complete subgraphs, Subject knowledge discovery, Computational biology and bioinformatics, Plant sciences

## Abstract

The potential discovery of protein complexes can elucidate the structure of protein-protein interaction networks and identify downstream regulatory genes. Given the complexity of protein-protein interactions, interpretable domain knowledge discovery has gained significant attention. In this study, we constructed a knowledge graph for interacting proteins by gathering data from UniProt and PlaPPISite databases related to the model plant Arabidopsis thaliana. We developed a relational subgraph-driven protein-protein interaction prediction model based on this knowledge graph to predict interactions within connected subgraphs. Subsequently, complete subgraphs of interacting proteins were extracted, enabling the potential discovery of protein complex structures. The knowledge graph consisted of 68,713 nodes and 109,496 semantic relationships. A total of 1,232 protein-protein interactions were predicted. Comparison with experimentally validated interactions recorded in the STRING and BioGrid databases revealed that 682 of these interactions were confirmed. Based on the predicted interactions, 336 protein complexes were identified by mining the complete subgraphs. The proposed knowledge mining method, which integrates relational subgraphs and complete subgraphs, facilitates the discovery of protein complexes and provides a novel approach for analyzing their structures and identifying downstream genes.

**Supplementary Information:**

The online version contains supplementary material available at 10.1038/s41598-025-18281-7.

## Introduction

In crop breeding research, proteins play critical roles in various life activities through interactions. Protein complexes have been identified as pivotal regulators of biological pathways^1^. As research progresses, an increasing number of protein complexes are being linked to key agronomic traits. These complexes regulate plant growth cycles and immune responses, protecting plants from diseases by recognizing and neutralizing pathogens. Thus, comprehensively exploring the functions and regulatory mechanisms of protein complexes is crucial for developing crop varieties that are more resilient to environmental changes and disease-resistant through genetic improvement.

In recent years, with the development of computational prediction and text mining techniques in bioinformatics, systematic methods for discovering protein interactions have gradually been established. Zhang et al. proposed a prediction method based on domain-domain interactions and protein-domain interactions^2^. Hou et al. utilized different attributes of protein sequences to predict protein interactions^3^; however, redundancy in sequence information complicates complete feature extraction. To address this, Devkota et al. proposed a network-based model integrating global and local diffusion embedding techniques to predict protein interactions by mining network structures^4^, although the accuracy was limited by network complexity. Li et al. introduced a deep integrated learning method to more accurately capture various features of proteins for interaction prediction^5^. These protein interaction prediction methods have laid a theoretical foundation for discovering crop protein complexes and provide valuable data resources for functional studies. However, existing prediction methods are often constrained by single-factor considerations, limiting their ability to comprehensively discover crop protein complexes.

Given the complexity of correlational relationships between biological entities, interpretable knowledge reasoning and discovery are particularly important. Knowledge graphs, which are graph-structured models representing semantic relationships, have proven effective for modeling biological entities and their intricate associations. The application of knowledge mining techniques for relationship reasoning has emerged as a promising approach for domain knowledge discovery^6,7^. Recent research on knowledge graphs has focused on single-step reasoning through representation learning and multi-step reasoning using relational paths. Models such as TransE, TransH, and TransR have demonstrated significant advantages in relational reasoning, offering powerful tools for uncovering novel associations between entities.

For example, Choi et al. extracted associations among chemicals, genes, diseases, and symptoms. Their study found that TransE, among knowledge representation learning models, performed best in discovering new associations and outperformed statistical inference methods based on mainstream databases^8^. However, TransE has limitations in handling complex relationships, such as one-to-many, many-to-one, and many-to-many associations. To address these challenges, advanced models such as TransH and TransR have been developed. Inspired by the TransH model, Wang et al. incorporated entity neighborhood information into knowledge representation learning, achieving improved predictive performance^9^.

Knowledge representation learning methods, including TransE and its derivatives, convert triple data into multidimensional vectors suitable for deep learning and neural network algorithms. However, this often reduces the interpretability of results and loses fine-grained semantic information. To overcome these limitations, researchers have explored combining graph models with knowledge representation learning. For instance, Wang et al. enhanced representation learning by integrating multi-level relational path features^10^, while Chen et al. used graph neural networks combined with TransE to consider both neighborhood and edge features^11^. As an effective clustering method, complete subgraphs represent specific organizational structures. By analyzing complete subgraphs in knowledge graphs, researchers can uncover potential relationships within the data, providing robust support for domain-specific knowledge discovery^12^.

Despite the development of protein interaction prediction models, mining protein complexes remains a formidable challenge. This study addresses this gap using knowledge graph technology to integrate multidimensional protein-related datasets. We propose a novel method for mining protein complexes by integrating relational subgraphs with complete subgraphs. First, a knowledge graph for interacting proteins is constructed. Then, a relational subgraph-driven protein interaction prediction model is developed to predict and complete missing protein-protein interactions. Finally, complete subgraphs of interacting proteins are extracted from connected subgraphs to facilitate the discovery of potential protein complexes.

## Materials and methods

### Data sources

In our research, the UniProt (Universal Protein Resource), and PlaPPISite databases^13^ were utilized as data sources. To address various breeding target levels with Arabidopsis thaliana as the research subject, such as growth and development, stress resistance, disease resistance, and economic indicators, five keywords were selected as “trait” entities: plant height, drought resistance, salt resistance, disease resistance, and insect resistance.

### Construction of a knowledge graph for interacting proteins

In the UniProt database, reviewed (Swiss-Prot) Arabidopsis proteins—manually curated and annotated—were selected to obtain multidimensional attribute information. Meanwhile, experimentally validated protein-protein interaction datasets were obtained from the PlaPPISite database. A multidimensional PPI knowledge graph was constructed with proteins as central entities.

### Retrieval of connected subgraphs for interacting proteins

A connected subgraph containing the query protein was retrieved from the knowledge graph using a breadth-first search algorithm. Starting from the query protein node, the algorithm marked traversed nodes and iteratively explored all neighboring nodes until no new neighbors were found. This method identified the maximum connected subgraph containing the input protein.

### Construction of a relational subgraph-driven protein-protein interactions prediction model

**Fig. 1:**
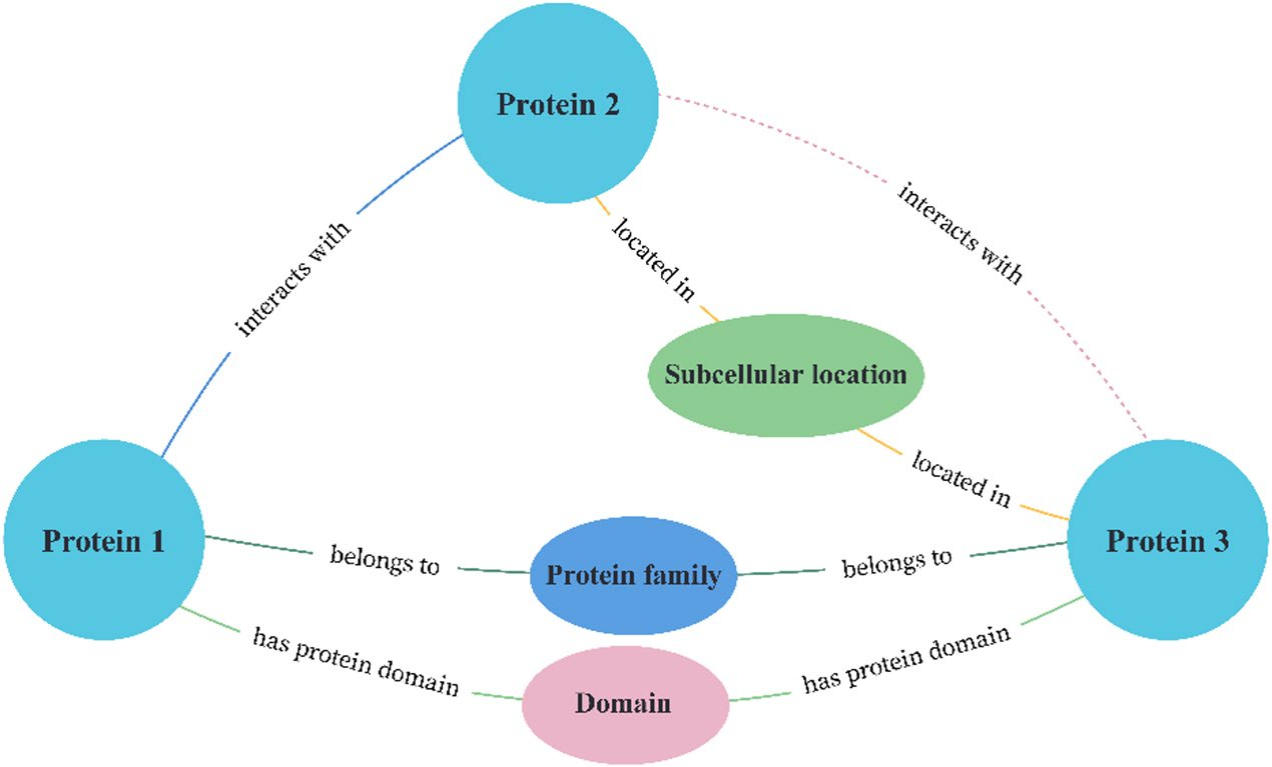
Relational subgraph-driven protein interaction prediction model.

A relational subgraph-driven protein-protein interaction prediction model was constructed using the knowledge graph. The model includes three node types: protein family, structural domain, and subcellular location. If protein 1 and protein 2 are known to interact, and protein 1 shares the same protein family or structural domain as protein 3, then protein 2 and protein 3 are predicted to interact. Additionally, proteins localized in similar subcellular locations are more likely to interact^14–16^. Based on these relationships, the model (Fig. 1) establishes the following rule:

- **IF** protein1 - [interacts with] - protein2.
- **AND** (protein1 - [belongs to] - protein family - [belongs to] - protein3 **or**.
- protein1 - [has protein domain] - domain - [has protein domain] - protein3)
- **AND** protein2 - [located in] - subcellular location - [located in] - protein3.
- **THEN** protein2 - [interacts with] - protein3.

### Mining for complete subgraphs in connected subgraphs

In order to supplement the missing edges in the connected subgraphs for interacting proteins, a protein-protein interactions prediction model was employed, which was based on the structure of the connected subgraphs for interacting proteins. In the completed connected subgraphs for interacting proteins, the Bron–Kerbosch maximum clique search algorithm was employed to mine the complete subgraphs for interacting proteins, identifying the largest complete subgraph containing the query protein. This facilitated the potential discovery of protein complexes.

To supplement missing edges in connected subgraphs, a relational subgraph-driven protein-protein interaction prediction model was used. The completed subgraphs were then analyzed using the Bron–Kerbosch maximum clique search algorithm^17^ to identify the largest complete subgraph containing the query protein. This enabled the discovery of potential protein complexes.

## Results

### Knowledge graph for interacting proteins

The model plant Arabidopsis thaliana, along with staple crops such as rice, maize, and wheat, were selected for scientific data collection, including all entities and their associated attributes. Using the described data sources and methods, triple data were stored in the non-relational graph database Neo4j, forming a knowledge graph for interacting proteins. The graph contained 68,713 nodes and 109,496 semantic relationships. We defied the semantic relationships and specific triple information according to the protein information (Table 1 and Supplementary Table S1).

**Table 1:** Statistics of triples in knowledge graph for interacting proteins.

Head entity; relation; tail entity	Head entity	Triples	Tail entity
(Gene; involves in; biological process)	12,365	12,365	6,683
(Gene; located in; cellular component)	13,701	13,701	3,644
(Gene; performs; molecular function)	12,424	12,424	3,802
(Protein; associates with; trait)	521	625	5
(Protein; belongs to; protein family)	12,858	12,858	2,522
(Protein; has protein domain; domain)	2,019	2,019	760
(Protein; identify with; gene symbol)	13,407	13,407	13,097
(Protein; interacts with; protein)	2,732	10,435	3,302
(Protein; involves in; signal pathway)	1,894	1,894	587
(Protein; is corresponding to; gene)	16,323	16,323	16,323
(Protein; located in; subcellular location)	12,511	12,511	4,885

### Connected subgraphs for interacting proteins

**Fig. 2:**
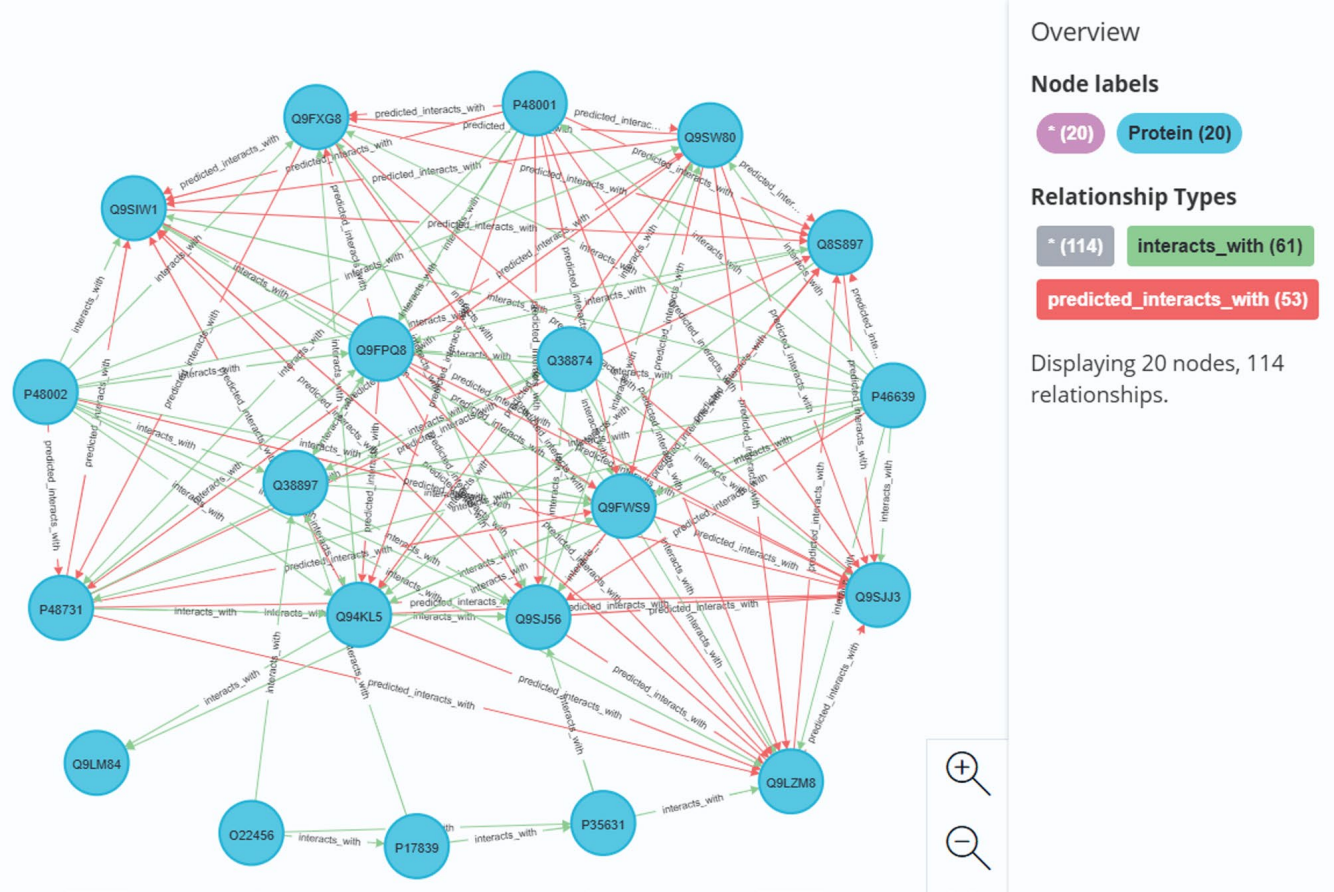
Schematic diagram of the connected subgraph for interacting proteins.

In the interacting protein knowledge graph, the protein connectivity subgraph reflects the dynamic functional units in the protein interaction network, which has potential functional synergy. Protein-protein interactions were predicted by multipath association within protein connectivity subgraphs to mine protein complete subgraphs. Therefore, on the basis of the knowledge graph of interacting proteins, the interacting protein connectivity subgraph was mined, and the connected subgraph containing the mined complete subgraph was selected as an example.These subgraphs contained complete subgraphs, which were retrieved by querying the knowledge graph with specified proteins (e.g., Q38874, Q9FPQ8, Q94KL5, Q9FWS9, Q9SJ56, or Q38897). One connected subgraph was found to contain 20 nodes, and a schematic representation of this subgraph is provided (Fig. 2).

### Prediction of protein-protein interactions

**Fig. 3:**
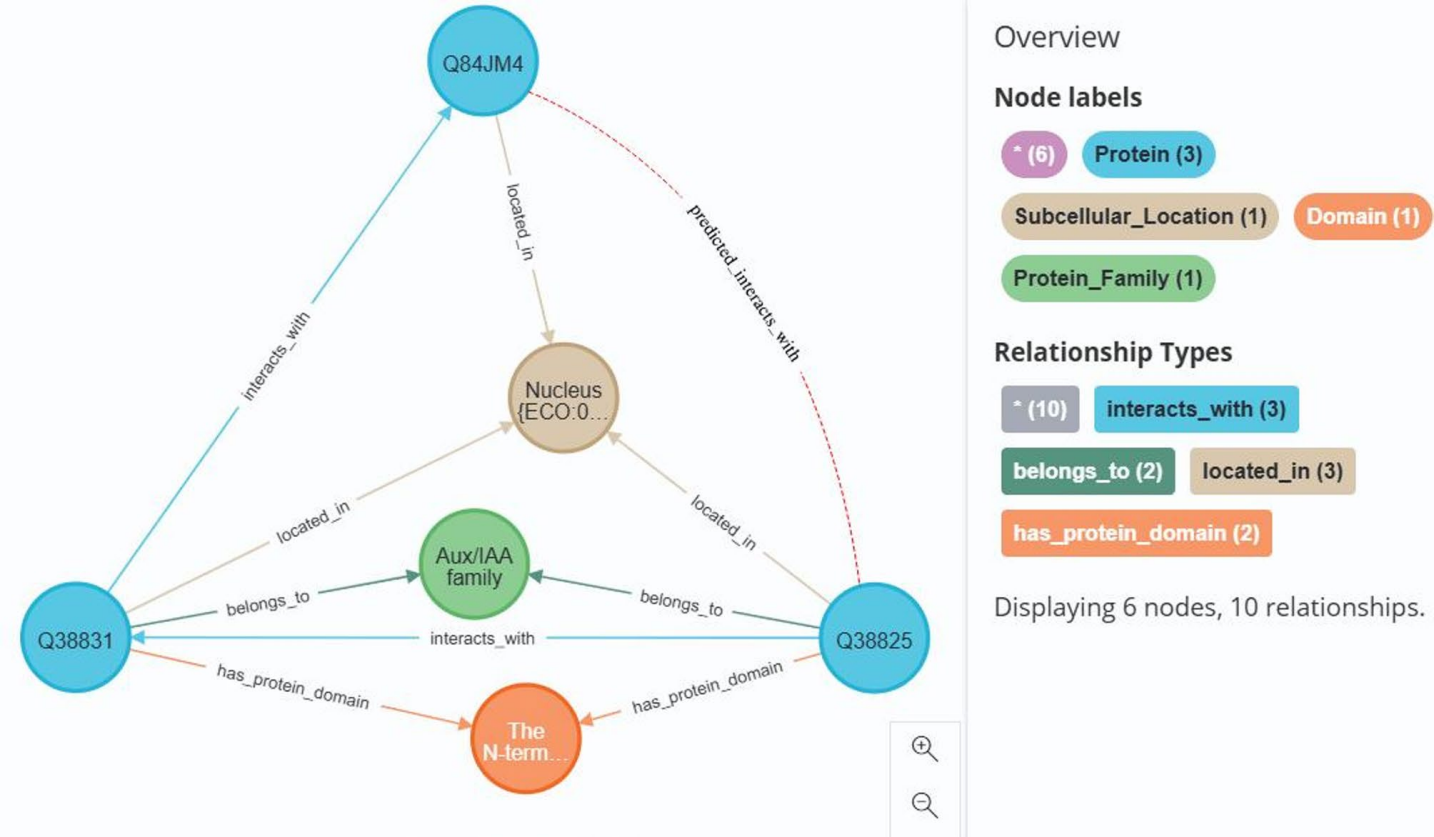
Prediction of the interaction between protein Q84JM4 and protein Q38825.

A relational subgraph-driven prediction model was employed to supplement and predict interactions within the connected subgraphs. This model successfully predicted 1,232 pairs of interacting proteins (Supplementary Table S2). Among these, 298 pairs overlapped with known protein interaction edges from the knowledge graph, while the remaining 934 pairs represent newly predicted interactions. For instance, protein Q84JM4 is known to interact with protein Q38831. protein Q38831 shares co-linked domain architectures and protein family associations with protein Q38825, while Q84JM4 exhibits co-linked subcellular localization patterns with Q38825. Based on our protein interaction prediction model, we propose a novel interaction between Q84JM4 and Q38825 (Fig. 3). This prediction aligned with known interactions in the knowledge graph, demonstrating the validity of the model. In addition, the identification of 934 novel protein-protein interactions provides a crucial data foundation for prospective discovery of protein complexes in subsequent research.

### Mining for protein complexes

In the mined connected subgraphs, the complete subgraphs of interacting proteins were further mined based on the network topology, and a total of 336 complete subgraphs containing the predicted edges of protein interactions were mined (Supplementary Table S3).

**Fig. 4:**
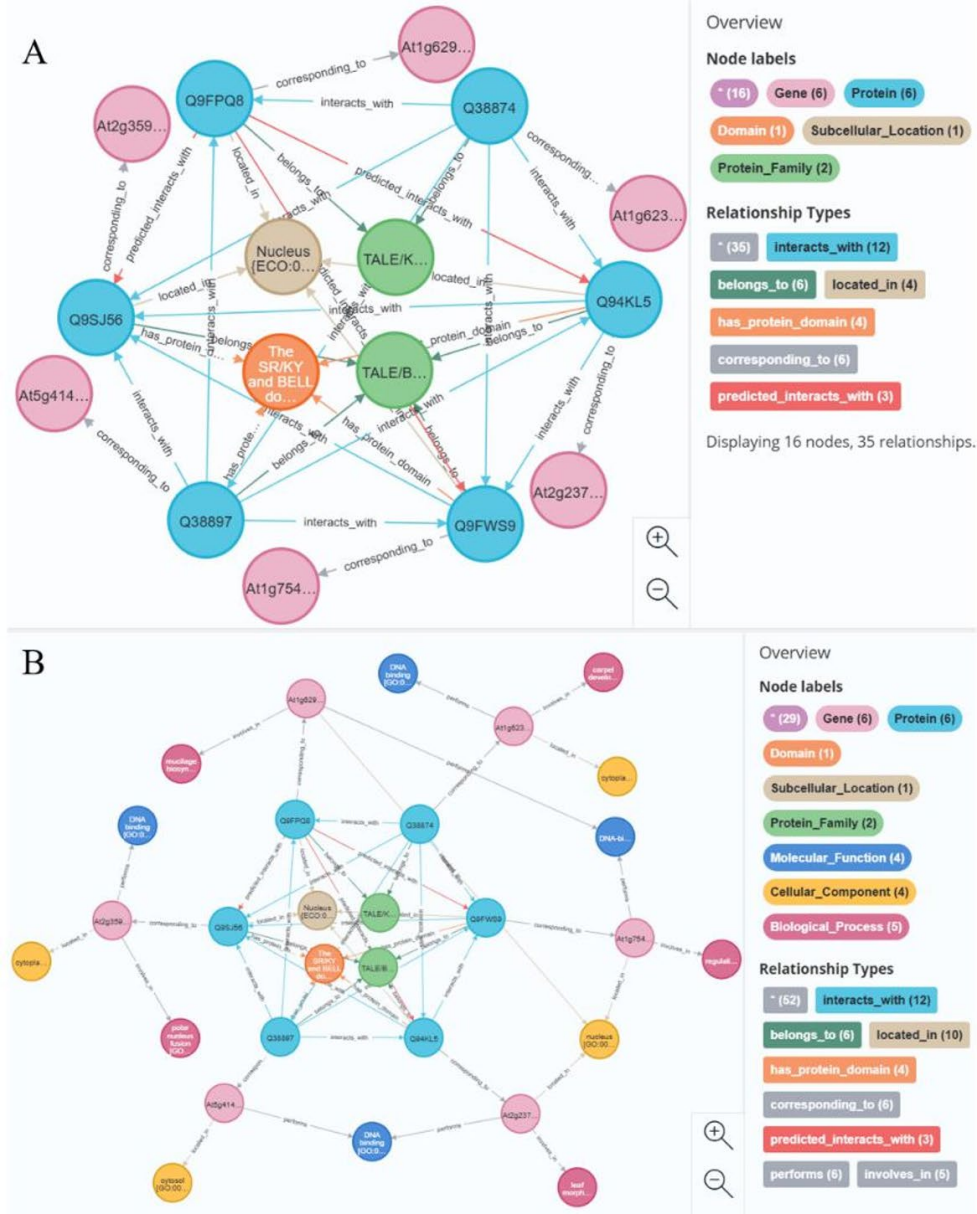
Potential discovery of protein complex Q38874-Q9FPQ8-Q94KL5-Q9FWS9-Q9SJ56-Q38897.

The completely connected subgraph involving Q38874, Q9FPQ8, Q94KL5, Q9FWS9, Q9SJ56, and Q38897 was selected for detailed illustration. Protein Q9FPQ8 is known to interact with Q38897. Q38897 and Q9FWS9 share co-linked protein family and domain nodes, while Q9FPQ8 and Q9FWS9 are associated via co-linked subcellular localization. Based on the relational subgraph-driven prediction model, new interactions between Q9FPQ8 and Q9FWS9, Q9SJ56 and Q9FPQ8, and Q9FPQ8 and Q94KL5 were predicted. These new interactions enabled the mining of a complete subgraph, leading to the discovery of a potential protein complex composed of these six proteins (Fig. 4A). Further multidimensional annotations were conducted for the mined protein complexes (Fig. 4B). Such findings enhance our understanding of how protein complexes coordinate biological processes at the molecular level, offering potential targets for genetic engineering to improve crop resilience and yield.

These novel complexes may play pivotal roles in signaling pathways or stress response networks. provide new insights into the molecular mechanisms underlying crop disease resistance, stress responses, and growth and development.

### Validation of mining results

**Fig. 5:**
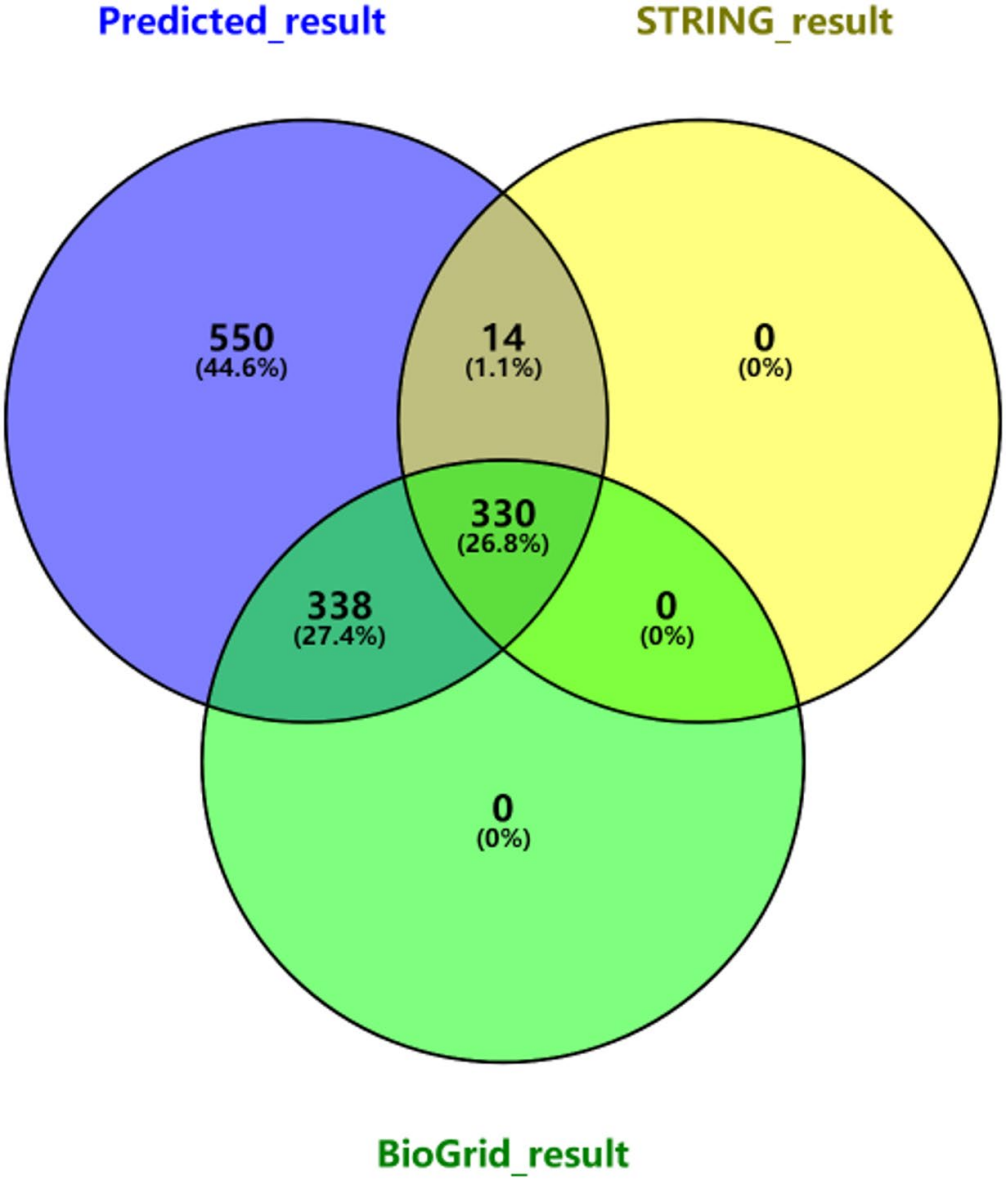
Venn diagram analysis of protein-protein interaction prediction outcomes.

In this paper, the potential discovery of protein complexes is realized based on the knowledge mining method of relational subgraph and complete subgraph fusion. A total of 1232 pairs of protein interactions were predicted by the protein-protein interaction prediction model. In order to further verify the scientific validity of the model, the protein interactions predicted in this study were compared with the experimentally validated protein interactions included in the BioGrid database^18^ and the STRING database^19^, of which 344 pairs of interacting proteins were validated by the STRING database and 668 pairs of interacting proteins were validated by the BioGrid database (Fig. 5). Finally, a total of 682 pairs of interacting proteins were experimentally verified. On the basis of the prediction results of interacting proteins, a total of 336 protein complexes were mined based on complete subgraphs.

## Discussion

Existing models for predicting protein-protein interactions, which are based on protein sequences^3^, structural domains^20^, or three-dimensional structures^21^, are often limited to single-dimensional analyses. These approaches are insufficient for exploring potential protein complexes due to their inability to capture the multidimensional nature of protein interactions. To overcome the limitations of single-dimensional inference, this study leveraged the semantic richness of knowledge graphs to build a multi-path, multi-factor integrated protein interaction prediction model, considering entities such as domains, protein families, and subcellular localization. A breadth-first search (BFS) algorithm was used to extract connected subgraphs, and the Bron–Kerbosch algorithm was employed to mine complete subgraphs. Ultimately, the discovery of potential protein complex structures was achieved by integrating relational and complete subgraphs. Validation of the model using existing databases demonstrated its effectiveness and highlighted its potential as a solution for protein complex mining.

The protein interaction prediction model constructed in this study leverages multiple inference paths to form relational subgraphs for protein interactions. This approach provides critical data for hypothesizing relationships between proteins using multipath evidence. For instance, searches using specific proteinIDs or pairs of proteinIDs generate multi-path association results, enabling a detailed analysis of potential interactions. Compared to traditional prediction methods, such as protein sequence feature extraction^22^, domain-based computational approaches^15^, or structure-based methods^23^, this model provides richer semantic information and enhances reasoning capabilities for protein-protein interactions.

Relational subgraph reasoning, as implemented in this study, addresses the challenges of interpretability associated with representation learning methods. It also mitigates errors inherent in single-path reasoning by leveraging multi-path association data. By constructing relational subgraphs, the proposed model provides a robust semantic foundation for predicting interactions and facilitates the discovery of potential protein complexes.

The fusion of relational subgraphs and complete subgraphs represents a significant methodological innovation in mining protein complexes. Mapping the complete subgraph structures within the knowledge graph to the network of interacting proteins allows for systematically identifying protein complexes. This approach not only improves the interpretability of protein-protein interaction predictions but also supports downstream applications, such as the identification of regulatory genes and pathways. For instance, the application of this method revealed seven novel protein complex structures that were not previously documented in existing databases, underscoring its potential for advancing knowledge in this domain.

In conclusion, the proposed method bridges the gap between single-factor protein interaction models and the need for comprehensive, multidimensional analyses. By integrating relational reasoning with graph-based methodologies, this study offers a novel framework for protein complex discovery, paving the way for further advancements in molecular biology and crop breeding research. Our study can analyze the specific regulatory mechanism of protein complexes in plant growth and development and stress resistance, and provide new targets for crop genetic improvement, providing directions for experimental validation and practical guidance for improving crop yields and enhancing disease and stress resistance. Nevertheless, the method may overlook protein complexes formed through interactions such as disulfide or hydrogen bonds. Additionally, current validations heavily rely on experimental data available in existing databases, and some predicted complexes remain experimentally unverified. Of course, in-depth research will be carried out in the following areas in the future. First, the influencing factors in the protein-protein interaction prediction model were optimized, and the protein post-translational modification and metabolomic data were further integrated to improve the prediction accuracy. Second, this study focuses on the model plant Arabidopsis thaliana, which can be extended to other important crops in the future to verify the universal adaptation of this method. Thirdly, the protein-protein interaction relationship was further verified through experimental methods, such as yeast two-hybrid and co-immunoprecipitation, and the specific regulatory mechanism of protein complexes in plants was analyzed.

## Conclusions

In summary, this study, using Arabidopsis thaliana as the model organism, constructed knowledge graph for interacting proteins comprises 68,713 nodes and 109,496 semantic relationships.

Based on this knowledge graph, a protein-protein interaction prediction model driven by relational subgraph was constructed. Furthermore, a protein complex mining model was constructed based on the fusion of the protein interaction prediction relationship subgraph and the protein interaction complete subgraph. Eventually, the potential discovery of protein complexes was realized.

This study demonstrates that integrating relational subgraphs and complete subgraphs offers a novel methodological framework for mining protein complexes. The approach not only supports multipath evidence-seeking analysis but also enhances the interpretability of protein-protein interaction predictions. These advancements have implications for the structural analysis of protein complexes and the identification of downstream regulatory genes. By providing an innovative and effective solution to the challenges of protein complex mining, this study gives an insight into cellular machinery and can help research community to identify underlying causes of crop diseases or suggest new targets for genetic modifications.

## Supplementary Information

Below is the link to the electronic supplementary material.

Supplementary Material 1

Supplementary Material 2

Supplementary Material 3

Supplementary Material 4

## Data Availability

The datasets analysed during the current study are available in the UniProt (universal protein resource) and PlaPPISite (a comprehensive resource for plant protein-protein interaction sites). The accession numbers were listed in the supplementary Table S4 (Supplementary Table S4).

## Code availability

The code presented in the study are included in the article/Supplementary. Further inquiries are available on request from the corresponding author.

## References

[1] Guo, Y. Z. et al. A novel method to predict protein-protein interactions based on the information of protein-protein interaction networks and protein sequence. *PPL* **18**, 906–911 (2011). 10.2174/092986611796011482 21529343

[2] Zhang, X., Jiao, X., Song, J. & Chang, S. Prediction of human protein–protein interaction by a domain-based approach. *J. Theor. Biol.* **396**, 144–153 (2016). 26925814 10.1016/j.jtbi.2016.02.026

[3] Hou, Q., De Geest, P. F. G., Vranken, W. F., Heringa, J. & Feenstra, K. A. Seeing the trees through the forest: sequence-based homo- and heteromeric protein-protein interaction sites prediction using random forest. *Bioinformatics* **33**, 1479–1487 (2017). 28073761 10.1093/bioinformatics/btx005

[4] Devkota, K., Murphy, J. M. & Cowen, L. J. GLIDE: combining local methods and diffusion state embeddings to predict missing interactions in biological networks. *Bioinformatics* **36**, i464–i473 (2020). 32657369 10.1093/bioinformatics/btaa459 PMC7355260

[5] Li, F., Zhu, F., Ling, X. & Liu, Q. Protein interaction network reconstruction through ensemble deep learning with attention mechanism. *Front. Bioeng. Biotechnol.* **8** (2020). 10.3389/fbioe.2020.00390 PMC7215070 32432096

[6] Lan, Y. et al. Path-based knowledge reasoning with textual semantic information for medical knowledge graph completion. *BMC Med. Inf. Decis. Mak.* **21**, 335 (2021). 10.1186/s12911-021-01622-7 PMC8628388 34844576

[7] Yang, R. et al. Decision-making system for the diagnosis of syndrome based on traditional Chinese medicine knowledge graph. *Evid.-Based Complement. Altern. Med.* **2022**, 1–9 (2022). 10.1155/2022/8693937 PMC8853781 35186106

[8] Choi, W. & Lee, H. Inference of biomedical relations among chemicals, genes, diseases, and symptoms using knowledge representation learning. *IEEE Access.* **7**, 179373–179384 (2019).

[9] Wang, Y., Wumaier, A., Sun, W., Liu, Y. & He, J. TransH-RA: A learning model of knowledge representation by hyperplane projection and relational attributes. *IEEE Access.* **11**, 29510–29520 (2023).

[10] Wang, Y., Zhao, E. & Wang, W. A. Knowledge graph completion method based on fusing association information. *IEEE Access.* **10**, 50500–50507 (2022).

[11] Web Information Systems Engineering – WISE 2019: 20th International Conference, Hong Kong, China, January 19–22, 2020, Proceedings. vol. 11881. (Springer International Publishing, 2019).

[12] Fang, L., Zhai, M. & Wang, B. Complete subgraphs in connected graphs and its application to spectral moment. *Discrete Appl Math.* **291**, 36–42 (2021).

[13] Yang, X. et al. PlaPPISite: a comprehensive resource for plant protein-protein interaction sites. *BMC Plant. Biol.* **20**, 61 (2020). 32028878 10.1186/s12870-020-2254-4 PMC7006421

[14] Dong, S. & Provart, N. J. *Analyses of Protein Interaction Networks Using Computational Tools*. Vol. 1794, (eds Two-Hybrid, S. & Oñate-Sánchez, L.) 97–117 (Springer New York, 2018). 10.1007/978-1-4939-7871-7_7 29855953

[15] Singhal, M. & Resat, H. A domain-based approach to predict protein-protein interactions. *BMC Bioinform.* **8**, 199 (2007). 10.1186/1471-2105-8-199 PMC1919395 17567909

[16] Lewis, A. C. F., Saeed, R. & Deane, C. M. Predicting protein–protein interactions in the context of protein evolution. *Mol. BioSyst*. **6**, 55–64 (2010). 20024067 10.1039/b916371a

[17] Yang, M., Tian, Y., Chen, J. L., Mao, J. C. & Song, Y. Application of bron-kerbosch algorithm for discovery of basic formulas of traditional Chinese medicine. *Zhongguo Zhong Yao Za Zhi*. **37**, 3323–3328 (2012). 23397738

[18] Oughtred, R. et al. The biogrid database: A comprehensive biomedical resource of curated protein, genetic, and chemical interactions. *Protein Sci.* **30**, 187–200 (2021). 33070389 10.1002/pro.3978 PMC7737760

[19] Szklarczyk, D. et al. The STRING database in 2023: protein–protein association networks and functional enrichment analyses for any sequenced genome of interest. *Nucleic Acids Res.* **51**, D638–D646 (2023). 36370105 10.1093/nar/gkac1000 PMC9825434

[20] Mosca, R., Céol, A., Stein, A., Olivella, R. & Aloy, P. 3did: a catalog of domain-based interactions of known three-dimensional structure. *Nucl. Acids Res.* **42**, D374–D379 (2014). 24081580 10.1093/nar/gkt887 PMC3965002

[21] Northey, T. C., Barešić, A. & Martin, A. C. R. IntPred: a structure-based predictor of protein–protein interaction sites. *Bioinformatics* **34**, 223–229 (2018). 28968673 10.1093/bioinformatics/btx585 PMC5860208

[22] Hu, L. & Chan, K. C. C. Extracting coevolutionary features from protein sequences for predicting protein-Protein interactions. *IEEE/ACM Trans. Comput. Biol. Bioinf.* **14**, 155–166 (2017). 10.1109/TCBB.2016.2520923 26812730

[23] Singh, R., Park, D., Xu, J., Hosur, R. & Berger, B. Struct2Net: a web service to predict protein-protein interactions using a structure-based approach. *Nucleic Acids Res.* **38**, W508–W515 (2010). 20513650 10.1093/nar/gkq481 PMC2896152

